# Sequencing Degraded DNA from Non-Destructively Sampled Museum Specimens for RAD-Tagging and Low-Coverage Shotgun Phylogenetics

**DOI:** 10.1371/journal.pone.0096793

**Published:** 2014-05-14

**Authors:** Mandy Man-Ying Tin, Evan Philip Economo, Alexander Sergeyevich Mikheyev

**Affiliations:** Okinawa Institute of Science and Technology, Onna-son, Kunigami-gun, Okinawa, Japan; University of Florence, Italy

## Abstract

Ancient and archival DNA samples are valuable resources for the study of diverse historical processes. In particular, museum specimens provide access to biotas distant in time and space, and can provide insights into ecological and evolutionary changes over time. However, archival specimens are difficult to handle; they are often fragile and irreplaceable, and typically contain only short segments of denatured DNA. Here we present a set of tools for processing such samples for state-of-the-art genetic analysis. First, we report a protocol for minimally destructive DNA extraction of insect museum specimens, which produced sequenceable DNA from all of the samples assayed. The 11 specimens analyzed had fragmented DNA, rarely exceeding 100 bp in length, and could not be amplified by conventional PCR targeting the mitochondrial cytochrome oxidase I gene. Our approach made these samples amenable to analysis with commonly used next-generation sequencing-based molecular analytic tools, including RAD-tagging and shotgun genome re-sequencing. First, we used museum ant specimens from three species, each with its own reference genome, for RAD-tag mapping. Were able to use the degraded DNA sequences, which were sequenced in full, to identify duplicate reads and filter them prior to base calling. Second, we re-sequenced six Hawaiian *Drosophila* species, with millions of years of divergence, but with only a single available reference genome. Despite a shallow coverage of 0.37±0.42 per base, we could recover a sufficient number of overlapping SNPs to fully resolve the species tree, which was consistent with earlier karyotypic studies, and previous molecular studies, at least in the regions of the tree that these studies could resolve. Although developed for use with degraded DNA, all of these techniques are readily applicable to more recent tissue, and are suitable for liquid handling automation.

## Introduction

Over the past two centuries, museum collections have grown in size and importance. They hold indispensable records of past scientific investigations, and also act as biological diversity libraries [1]–[3]. Many species are most easily accessible in museums, either because of their rarity, their remote geographic distribution, the expertise required to identify them, or even because they have already gone extinct. Although museum collections have always held great value for morphology-based research, they have been underutilized as a genetic resource. The majority of material used to organize biodiversity in the last several centuries remains outside the scope of modern molecular genetics, including modern molecular phylogenetic efforts to reconstruct the tree of life. This is in part because archival specimens tend to be fragile and valuable. DNA extractions can be destructive, and curators are rightfully protective. Furthermore, since the vast majority of archived specimens contain badly degraded DNA, they are not suitable for the most commonly used methods of targeted PCR-based gene sequencing. If these challenges can be overcome, and these specimens can be brought into the era of molecular biology, it would open new research possibilities and would again place museum collections at the center of biodiversity research.

A number of protocols have been developed for non-destructive sampling of specimens, which can sometimes produce DNA suitable for PCR amplification of targeted genes [4]–[11]. These protocols typically focus on mitochondrial genes, which have high copy numbers. For the level of DNA degradation typically found in museum specimens, 35 or more PCR cycles were necessary to obtain products [5], [6], [9], [10]. This treatment potentially amplifies trace quantities of less degraded contaminating material present in the sample [12]. Furthermore, at a certain point DNA fragments become too small to detect using targeted PCR, because the maximum fragment size becomes shorter than the region of interest. Such specimens may still contain valuable genetic material, but it cannot be amplified by PCR, precluding conventional approaches.

Next-generation sequencing methods have been extensively employed in vertebrates, where a small fraction of a museum specimen can be destructively sub-sampled for sequencing library preparation (*e.g.* [13] and citations within). Being relatively large, such specimens generally yield considerable amounts of DNA. These studies have generally relied on commercially available kits for library preparation. To the best of our knowledge all such kits have an ‘end-repair’ step that uses 5’ exonuclease activity to retain only double-stranded DNA. This means not only the loss of information on the 5’ end of the DNA strand, but complete degradation of single-stranded DNA. While a new approach by Gansauge and Meyer [14] circumvents many of the pitfalls associated with end-repair, there is still room for improvement. For instance, it is sensitive to the concentration of starting material, and requires a low and narrow sample input range (between 13 pg and 13 ng), and necessitating a large number of PCR cycles [15]. While this may be inevitable for ancient DNA, where only trace quantities of material remain, a large number of cycles may introduce unnecessary PCR artifacts. Furthermore, the kinetics of single-stranded ligation limit maximum range of fragments preparable by this method to 120 bp [16]. Finally, the Gansauge and Meyer protocol is labor intensive and, because it relies on a specialized ligase, expensive, making it difficult to implement in a high-throughput manner, which would be necessary, for example, for population genetic studies.

**Figure 1:**
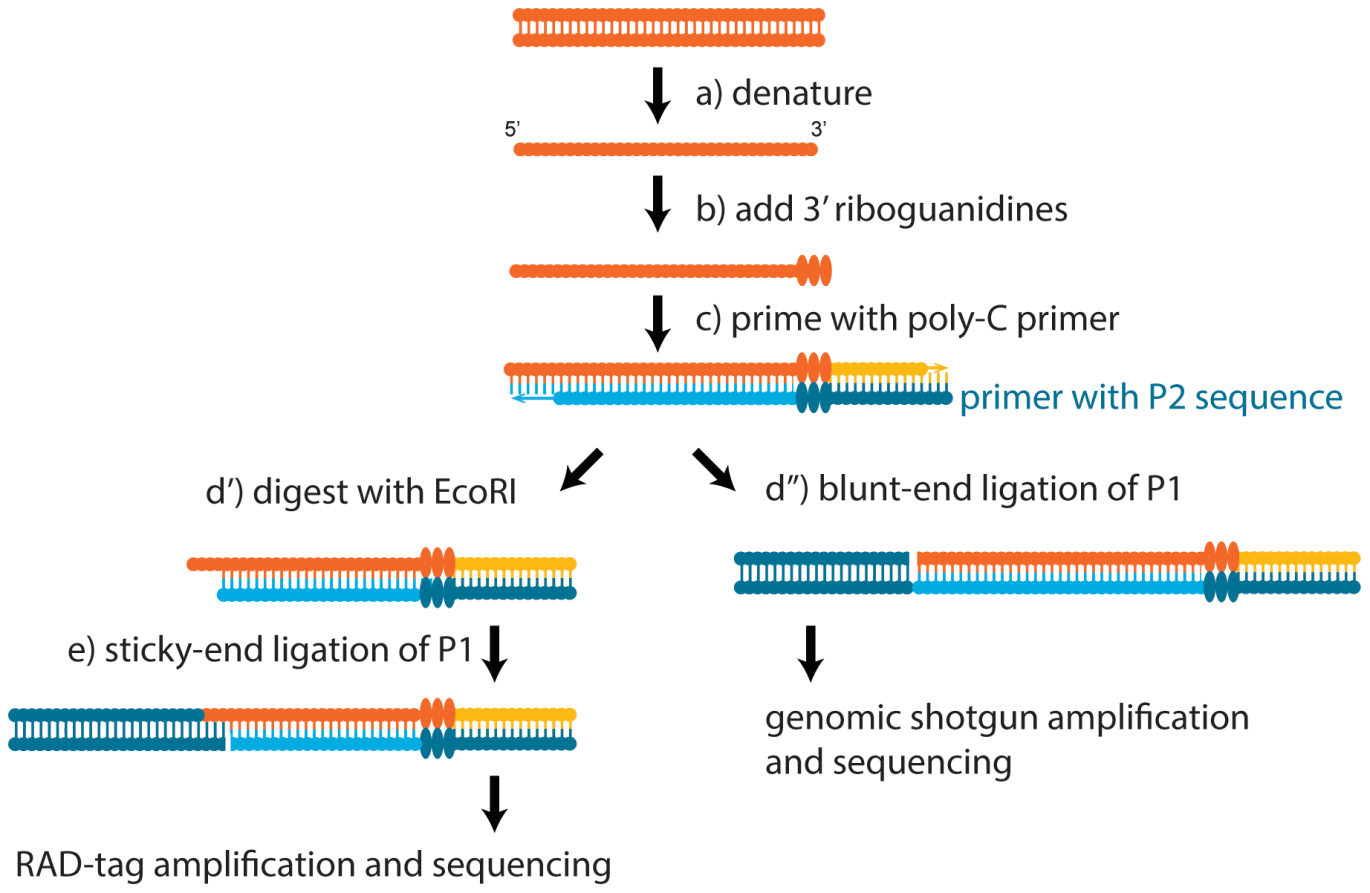
Schematic overview of the library preparation process. Both RAD-tag (left) and whole-genome shotgun (right) library preparation methods start the same way, and diverge only at the final stage. (a) DNA is heated to denature the template strands. (b) Terminal deoxynucleotidyl transferase (TdT) is used to add a riboguanidine tail of a determined length [44]. (c) Priming with the Illumina P2 adaptor sequence, the Klenow exo- fragment generates the second strand. At this point, T4 DNA polymerase treatment is necessary to blunt the DNA fragments. After (d’) for RAD-tag sequencing, EcoRI is used to digest a subset of the fragments. (d’’ and e) a final ligation step adds the P1 Illumina adaptor sequence. Barcodes are ligated in-line, upstream of the read one sequencing primer binding site. After ligation of the final adaptor sequence, fragments are PCR-amplified to complete the sequencing adaptor. All libraries contained in-line barcodes in front of the read one sequencing site.

Our goal was to develop a robust, automatable pipeline that to recover genomic data from badly degraded museum specimens, with the secondary objective of minimizing damage due to extraction. To this end, we conducted two experiments. First, we used vouchered specimens from three ant species with sequenced genomes to extract DNA for a low-coverage re-sequencing study using restriction-associated DNA tags (RAD tags) [17], [18]. This flexible technique has been used for a large number of applications, ranging from marker discovery, to whole genome association studies, to phylogenetics, to delineating species boundaries [19]–[21]. Because the RAD tag procedure relies on fragmented DNA, it should be well suited to analyzing degraded archival DNA. Second, we conducted whole-genome re-sequencing on vouchered Hawaiian fruit fly specimens, representing six species, for which there was only one reference genome. We were able to construct a well-resolved phylogeny obtained from low-coverage whole-genome sequence data acquired from degraded DNA. Conventional phylogenetic techniques, which require hundreds of bases of intact DNA would not have worked for these samples. These experiments show applications of our library preparation method to the analysis of archived specimens with modern sequencing tools. An overview of the molecular methods can be seen in Figure 1.

## Methods and Materials

### Ethics statement

Ken Kaneshiro (University of Hawaii Insect Museum) and L. Lacey Knowles (University of Michigan Museum of Zoology) loaned specimens for this study.

### Specimens used in this study

Six *Drosophila* specimens from six species (*anomalipes*, *grimshawi*, *hawaiiensis*, *paucipuncta*, *picticornis*, and *silvestris*) were collected between 1966–1976 in Hawaii and were stored in the University of Hawaii Insect Museum. Ant specimens were collected between the years 1910–1953 and are part of the University of Michigan Museum of Zoology (UMMZ) insect collection. For the ants we used two specimens each of *Camponotus floridanus* and *Pogonomyrmex barbatus* and one specimen of *Linepithema humile*. Both fruit flies and ants were taken from series comprising several representatives of the same species. There were no representatives of any of the species used in the laboratory prior to this study. The specimens were processed simultaneously, so there was no possibility of cross-contaminating extracts with post-PCR products.

### Genomic DNA extraction from museum specimens

The bench top was cleaned with bleach before extraction, then wiped clean with distilled water. Forceps were cleaned with ethanol and flamed before handling specimens. Only guaranteed DNA-free disposable consumables were used and reagents were dedicated to old DNA research only. Consumables and reagents were all separately from those used in other experiments in the lab. Filter tips were used for all experimental procedures. DNA extraction buffer contained 50 g guanidine isothiocyanate, 5.3 ml of 1 M Tris-HCl, pH 7.5, 5.3 ml of 0.2 M EDTA, 10.6 ml of 20% Sarkosyl (IBI Scientific) and 1 ml β-mercaptoethanol, dissolved in 50 ml water [9]. MilliQ-purified water was used at all steps in the experiment. Whole specimens were placed in 1.5 ml microcentrifuge tubes with 200 µl DNA extraction buffer for an overnight incubation at 55°C. Specimens were neither homogenized, nor in any way mechanically damaged. The point upon which the specimens were mounted was trimmed to fit into the extraction vial, but remained attached to the specimen (it detached in the course of treatment). An equal volume of 99.5% ethanol and 20 µl of silica magnetic beads (Chemicell GmbH) were added to the DNA lysate. Tubes were gently mixed and incubated on a rotary mixer at room temperature for 15 minutes. All chemicals were purchased from Nacalai Tesque, Inc., unless otherwise stated.

Tubes were then placed on a magnetic stand (Invitrogen) for 5 minutes to separate the supernatant from the magnetic beads. Supernatant that contained proteins and other impurities was discarded. Beads with bound DNA were washed with 200 µl PE buffer (Qiagen) for 10 minutes at room temperature on a rotary mixer followed by bead separation on a magnetic stand. The washing step was repeated two more times and beads were then air dried for 30–45 minutes. Tubes were then removed from the magnetic stand and DNA was eluted from the beads by re-suspending them in 40 µl EB. For *Drosophila* specimens, the elution volume was 20 µl. After 10 minutes incubation at 55°C, DNA was separated from the beads on a magnetic stand and transferred to new microcentrifuge tubes. The 260/280 ratio of the DNA was measured by Nanodrop (Thermo Scientific). DNA fragment size was evaluated with an Agilent High Sensitivity DNA kit (Agilent Technologies). The quantity of the DNA was estimated by Quant-iT PicoGreen dsDNA Assay Kit (Invitrogen).

### PCR amplification of a mitochondrial gene

The mitochondrial cytochrome c oxidase subunit I (COI) gene is a commonly used marker for phylogenetic and taxonomic analysis. It can be robustly amplified using the LCO1490/HCO2198 primer pair across different phyla including other species of *Drosophila* and in other ants [22], [23]. To investigate whether degraded DNA samples could be utilized with conventional sequencing methods, PCR was carried out in a 20 µl reaction consisting of 1x Ex-Taq buffer, 200 µM dNTP, 0.2 µM of each primer, 0.5 U TaKaRa Ex-Taq (Takara) and 1 µl of extracted DNA. The DNA extract of old specimens contained 9 – 23 ng of DNA, except for extracts of museum specimens of the ant, *Linepithema humile*, from which only about 1.5 ng of DNA was used for PCR (this species has by far the smallest body size of all species used). DNA extracted from recently preserved specimens was used as a positive control and about 10 ng DNA were employed. Reactions were carried out under the following conditions: initial denaturation at 94°C for 2 minutes, followed by 35 cycles of 20 seconds at 94°C, 1 minutes at 40°C and 1 minute 30 seconds at 72°C. Final extension was performed at 72°C for 5 minutes. 4 µl of PCR products were resolved by electrophoresis on a 1% agarose gel.

### RAD-seq of museum ant specimens

### Addition of guanosine 5’-triphosphates (GTP) to the 3’termini of genomic DNA

Since single-stranded DNA is more efficiently tailed with ribonucleoside 5’-triphosphates than is double-stranded DNA [24], 10 µl genomic DNA (between 14–220 ng in total) were heat denatured at 95°C for 10 minutes and then quickly chilled on ice before the ribo-tailing reaction with terminal transferase (TdT). The reaction consisted of 1x buffer 4 (New England Biolabs), 2.5 mM cobalt chloride (New England Biolabs), 4 mM GTP (Takara), 10 U TdT (New England Biolabs) and 10 µl denatured genomic DNA in 20 µl reaction volume. Reactions were incubated at 37°C for 30 minutes and then TdT was heat-inactivated at 70°C for 10 minutes.

### Second strand DNA synthesis

The microcentrifuge tube containing Dynabeads M-280 Streptavidin (Invitrogen) was placed on a magnetic stand and storage buffer was removed by aspirating the supernatant. Beads were washed twice in PBS with 0.1% BSA (Sigma), twice more in 2x binding and washing (B&W) buffer (10 mM Tris-HCl, pH 7.5, 1 mM EDTA and 2 M NaCl) and then resuspended in 2x B&W buffer to a final concentration of 5 µg/µl. One nmole of an oligonucleotide with the Illumina P2 sequence: 5’-Biotin-TCTCGGCATTCCTGCTGAACCGCTCTTCCGATCTCCCCC was immobilized on M-280 Dynabeads (Invitrogen) by combining with 500 µg prepared beads in a 1∶1 volume ratio. The mixture was incubated at room temperature for 15 minutes followed by bead separation on the magnetic stand. Beads were washed five times with water to remove excess adaptors and resuspended in 100 µl EB buffer (10 mM Tris, pH 8.5, Qiagen).

The Illumina P2 sequence was added to the riboguanine-tailed DNA by base pairing with homopolymeric deoxycytidine triphosphates of the oligo. Klenow Fragment (3′→ 5′ exo-) (New England Biolabs) was used to synthesize the complementary sequence of the Illumina P2 sequence and second strand DNA. After addition of 10 µl reaction mix, consisting of 1 µl of 10x buffer 4, 0.6 µl of 25 mM dNTP (Promega), 5 µl of Illumina P2 oligo beads and 10 U Klenow Fragment (3′→ 5′ exo-), to the TdT reaction mix, the mixture was incubated at room temperature for 3 hours with constant rotation. Supernatant was separated from the beads on a magnetic stand and discarded. Beads were washed two times with 20 µl water.

### EcoRI digestion and ligation of Illumina P1 adaptor with in-line barcodes

Digestion and ligation were carried out on double-stranded DNA fragments bound to the beads. The reaction consisted of 1.4 µl of 10x T4 ligase buffer (New England Biolabs), 1.3 µl of 0.5 M sodium chloride (NaCl), 0.65 µl of 1 mg/ml bovine serum albumin (New England Biolabs), 5 U EcoRI (New England Biolabs), 80 U T4 DNA ligase (New England Biolabs) and 0.5 µl of 50 µM adaptor (top:5’-AATGATACGGCGACCACCGAGATCTACACTCTTTCCCTACACGACGCTCTTCCGAT-CTxxxxx [xxxxx = barcode]; bottom: 5’-AATTxxxxxAGATCGGAAGAGCGTCGTGTAGGGAAA), in a final reaction volume of 13 µl. Non-phosphorylated adaptors were used to reduce non-specific ligation. After 2 hours incubation at 37°C, supernatant was separated from beads on a magnetic stand and discarded. Beads were washed twice with 20 µl water, and then resuspended in 10 µl EB buffer. 1 µl of the bead suspension was used in 50 µl PCR amplification with 1x Phusion HF buffer (Thermo Scientific), 200 µM dNTP, 0.5 µM P1 primer: AATGATACGGCGACCACCGAGATCTACACTC, 0.5 µM P2 primer: CAAGCAGAAGACGGCATACGAGATCGGTCTCGGCATTCCTGCTGAACCGCTCTTCCGATCT, and 1 U Phusion DNA Polymerase (Thermo Scientific). PCR was performed under the following conditions: initial denaturation at 98°C for 30 seconds was followed by 25 cycles of denaturation at 98°C for 10 s, annealing/extension at 72°C for 30 seconds with a final extension step at 72°C for 5 minutes.

### Purification of RAD-tag libraries

Dynabeads MyOne Carboxylic Acid (Invitrogen) were washed twice in EB buffer (10 mM Tris-Cl at pH 8.5) and then resuspended in the same volume of EB buffer. We used a range of polyethylene glycol (PEG) 6000 concentrations for DNA precipitation. Each was dissolved to the desired percentage with a final concentration of 0.9 M NaCl and 10 mM Tris, pH 6. 100 µl of 14.5% PEG-6000/NaCl/Tris and 10 µl of prepared Dynabeads were added to each PCR and resuspended. The mixture was incubated at room temperature for 5 minutes. Tubes were then placed on a magnetic stand for 5 minutes. Supernatant was discarded and beads, which would have captured DNA longer than approximately 100 bp, were saved. Beads were washed twice with 70% ethanol (with 10 mM Tris, pH 6 in final concentration), and dried for 5 minutes at room temperature. Tubes were then taken off the magnetic stand and bound DNA was eluted from the beads by resuspending them in 15 µl EB buffer. After 5 minutes incubation at room temperature, beads were separated from DNA on the magnetic stand. The concentration of DNA was measured with Quant-iT PicoGreen dsDNA Assay Kit (Invitrogen). Equimolar concentrations of libraries with different barcodes (5’-ATCAC, 5’-CGATC, 5’-CCGTAC, 5’-TATCTCC, 5’-TATGCTAC) were pooled. The 6-plex libraries were purified again with 15.5% PEG-6000/NaCl/Tris and Dynabeads.

### Genomic shotgun library preparation of Hawaii *Drosophila* specimens

Procedures for library preparation were the same as described above, but with three modifications. First, 300 ng genomic DNA were used. Second, an unbiotinylated oligo with the same sequence was used for the Klenow Fragment (3′→ 5′ exo-). Third, in order to remove excess oligo from the previous reaction prior to ligation, the mixture was purified with MinElute Reaction Cleanup Kits (Qiagen), rather than with magnetic beads.

### Second strand synthesis

DNA was heat-denatured to single-stranded DNA at 95°C for 10 minutes and then quickly chilled on ice. An rG tailing reaction was performed and heat-inactivated. 10 µl of reaction mix, consisting of 1 µl 10x NEB Buffer 4, 0.6 µl of 25 mM dNTP, 1 µl of 15 µM Illumina P2 oligo and 10 U Klenow Fragment (3′→ 5′ exo-) were added to the TdT reaction mix. The mixture was incubated at room temperature for 3 hours. The enzyme was inactivated at 75°C for 20 minutes. Klenow Fragment (3′→ 5′ exo-) tends to leave a single base 3’ overhang. T4 DNA polymerase (New England Biolabs) was used to create blunt-end DNA fragments. 5 µl DNA blunting reaction mix consisted of 0.5 µl 10 x buffer 4, 0.35 µl of 10 mg/ml BSA, and 0.6 U T4 DNA polymerase were then added to each reaction. Reactions were incubated at 12°C for 15 minutes and purified with MinElute Reaction Cleanup Kit (Qiagen).

### Ligation of Illumina P1 adaptor with in-line barcodes

Libraries were constructed by ligation of blunted DNA fragments with Illumina P1 adaptors. The 20 µl reaction consisted of 1 x T4 DNA ligase buffer, 0.5 µl of 50 µM adaptor (top: 5’-CGACGCTCTTCCGATCTxxxxxxddC [xxxxxx  =  barcode; ddC  =  2’, 3’-dideoxycytidine triphosphate]; bottom: 5’-phosphate-GxxxxxxAGATCGGAAGAGCGTCGTGTAGGGAAAGAG-TGTAGATCTCGGTGGTCGCCGTATCATT), 1 µl of 400,000 cohesive end unit/ml T4 DNA ligase, and the blunted DNA. The ligation reaction was carried out at 16°C overnight.

3 µl ligation reaction were used as template for PCR amplification with 1x Phusion HF buffer (Thermo Scientific), 200 µM dNTP, 0.5 µM P1 primer: AATGATACGGCGACCACCGAGATCTA-CACTC, 0.5 µM P2 primer: CAAGCAGAAGACGGCATACGAGATCGGTCTCGGCATTCCTGC-TGAACCGCTCTTCCGATCT, and 0.6 U Phusion DNA Polymerase (Thermo Scientific). PCR was carried out in 30 µl final volume with the following conditions: initial denaturation at 98°C for 30 seconds was followed by 17 cycles of denaturation at 98°C for 10 s, annealing/extension at 72°C for 30 seconds, with a final extension step at 72°C for 5 minutes.

### Purification of genomic libraries prior to sequencing

PCR products were adjusted to 50 µl final volume with MilliQ-purified water before purification. 17% PEG-6000/NaCl/Tris and Dynabeads were used to purify the PCR reactions. Concentrations of DNA were measured with Quant-iT PicoGreen dsDNA Assay Kit. Equimolar concentrations of libraries with different barcodes (5’-GAGGAT, 5’-GTCCAA, 5’-AGATT, 5’-ATCAC, 5’-TCAT and 5’-CGT) were pooled. The 6-plex libraries were purified again with 19% PEG-6000/NaCl/Tris and Dynabeads, as described above for RAD-tag library purification.

### Sequencing of libraries

All libraries were analyzed with a Bioanalyzer High Sensitivity DNA Kit. Quantitative PCR (KAPA Biosystems) was used to estimate DNA concentrations of the libraries. Ant libraries were sequenced singled-ended for 50 cycles using the Illumina MiSeq system (control software version 2.1). Fruit fly libraries were sequenced on the same instrument, but after the manufacturer’s upgrade to version 2.2, and using 100-cycle single-end reads. We then exhaustively sequenced the fly libraries using two lanes of the HiSeq 2500 in rapid cycling mode.

### Genome mapping and phylogenetic analysis for fruit flies

Raw reads were sorted by barcode using grep regular expressions to match exact substring sequences, and filtered to trim adaptors from 3’ ends of the reads. We observed an excess of 5’ cytosines in fly genomic shotgun libraries, which were trimmed prior to read mapping. Because short reads can map ambiguously, we filtered reads shorter than 20 bp. Read trimming and filtering by length was carried out with cutadapt (v1.2.1) [25]. Filtered reads were mapped to the reference genome using bowtie2 (b2.0.5) [26]. We used the official assemblies of the ant and *Drosophila grimshawi* genomes as mapping references [27]–[30]. After mapping, we removed reads that were probable PCR duplicates using Picard tools. Single nucleotide polymorphisms (SNPs) were inferred using the GATK pipeline, following the Broad Institute’s best practices guidelines, including base and variant quality score recalibration [31], for which we used samtools as an additional base caller [32]. In order to avoid sites within repetitive elements, we annotated them using RepeatMasker [33], and masked them using bedTools [34]. The resulting SNP set was further reduced to included only sites that were present in at least 4 of the 6 genomes. We performed phylogenetic inference using MrBayes (v3.2.1) using a discrete character model for SNPs, while assuming a gamma rate heterogeneity among sites [35]. SNPs that were not fixed within species were eliminated from the analysis. Our analysis was computed using two MCMC runs of 1,000,000 generations each with a 25% burn-in using four chains for each run, and setting *D. anomalipes* as the outgroup.

### Genome mapping and RAD-tag analysis for ants

We followed the same approach for mapping ant RAD-tags, as we did fruit fly genomic shotgun reads, except that the mapping criteria were slightly relaxed, given the shorter length of the read. In addition, since there were not enough markers to conduct reliable quality score recalibration, here we present the raw GATK output, which should be taken with a degree of skepticism. Because the vast majority of DNA fragments were shorter than the Illumina read size, we could use the end of the read to distinguish between individual fragments and to count duplicates.

### Code availability

The bioinformatic and statistical analysis pipeline can be found at https://github.com/mikheyev/DNA-repair, along with the raw output from the major steps in the analysis.

## Results

### Specimen extraction and genomic DNA quality

**Figure 2:**
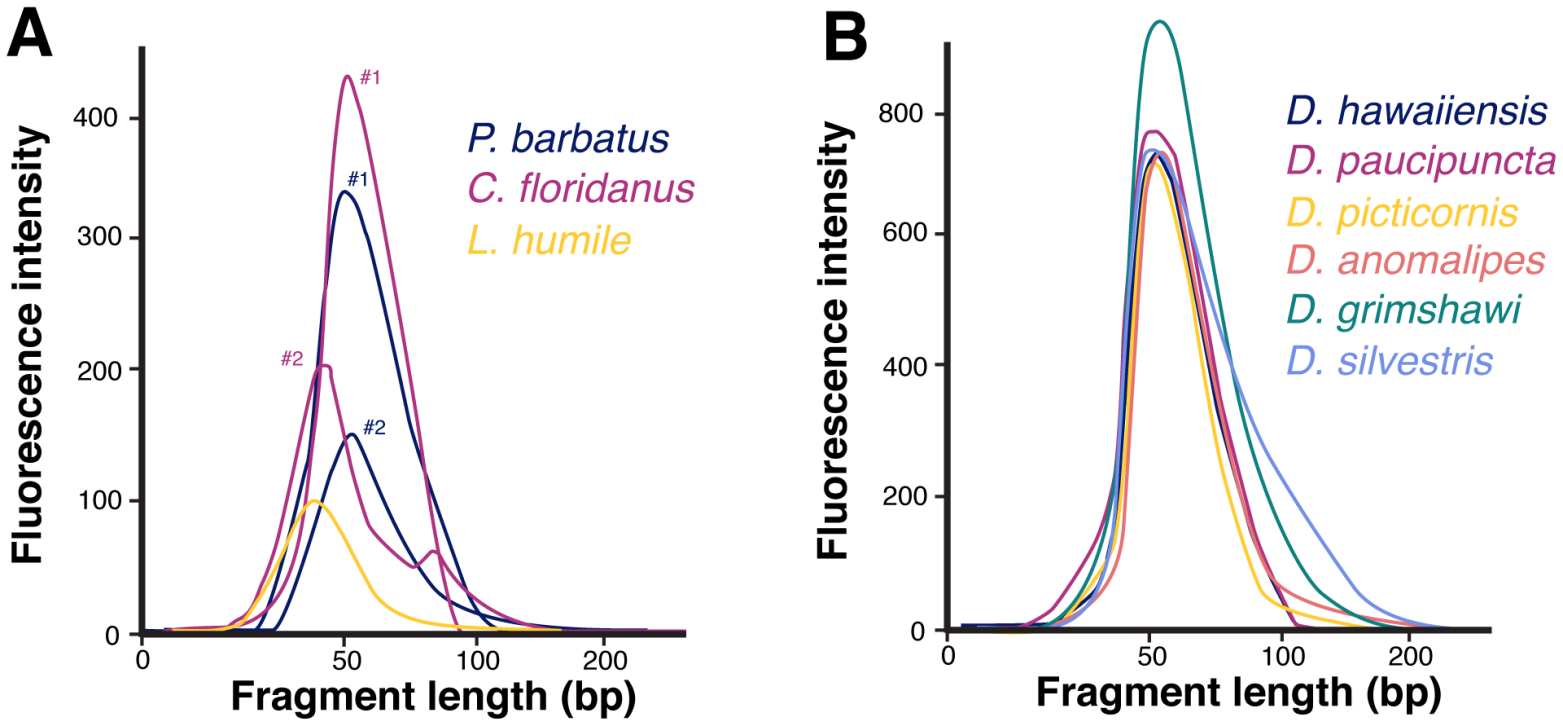
Fragment sizes of extracted DNA for ants (A) and fruit flies (B). Virtually all fragments in the libraries were less than 100-axis are derived from Bioanalyzer traces, and are somewhat arbitrary, giving only an approximate indication of extraction yield. See Table 1 for more details on the actual yield from each extraction. We failed to amplify an approximately 700-bp fragment of mitochondrial DNA from these specimens using a popular primer set [22].

All specimens produced significant DNA yields (Table 1). The DNA obtained by the protocol was relatively pure of contaminants, particularly protein, as measured by the ratio of absorbances at 260/280 nm (1.7±0.1), close to a ‘pure’ DNA value of 2.0. However, the DNA was significantly degraded. Mean fragment sizes were 55±14 bp in ant samples, and 66±3 bp in fruit fly samples (Figure 2). None of the specimens could be amplified using a common primer set, targeting a large (∼700 bp), high copy fragment of COI with universal primers commonly used in DNA barcoding.

**Table 1:** Specimens used in this study, and DNA yields.

Specimen id	Year	DNA yield (ng)	260/280 ratio	DNA used (ng)	Filtered reads	Mapped read %	Duplicate read %
*P. barbatus* #1	1920	766	1.64	219	2.8×10^5^	76	47
*P. barbatus* #2	1953	387	1.54	111	6.0×10^5^	56	36
*C. floridanus* #1	1953	720	1.7	206	3.4×10^5^	74	45
*C. floridanus* #2	1910	320	1.65	92	5.9×10^4^	72	40
*L. humile*	1920	50	1.43	14	9.2×10^4^	74	44
*D. anomalipes*	1968	515	1.77	300	3.3×10^7^	64	56
*D. grimshawi*	1968	742	1.76	300	2.9×10^7^	53	45
*D. hawaiiensis*	1966	463	1.74	300	2.5×10^7^	32	29
*D. paucipuncta*	1968	483	1.65	300	1.1×10^7^	19	15
*D. picticornis*	1968	423	1.73	300	1.8×10^7^	33	29
*D. silvestris*	1976	546	1.7	300	4.1×10^7^	58	48

See Figure 2 for data on the fragment size distribution in the libraries. The number of reads is much higher for the *Drosophila* libraries because they were sequenced exhaustively on a HiSeq, as well as a MiSeq platform.

**Figure 3:**
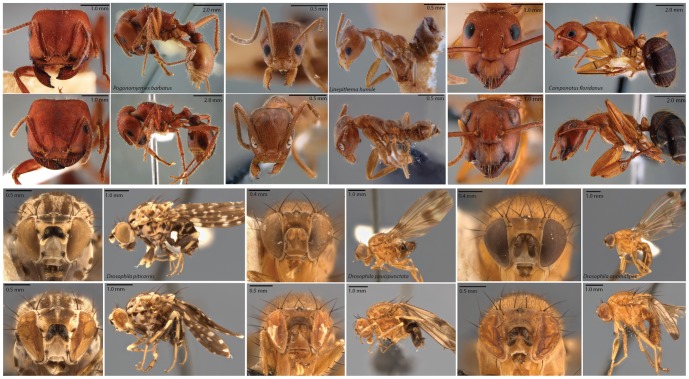
Images of representative specimens before and after extraction. Specimens before extraction are in first and third rows, and after extraction in second and fourth rows. For ant photos, the same specimen is depicted before and after extraction. For *Drosophila*, different specimens from the same collection series were used for the before-and-after comparison. Specimen damage to ant was minimal, consisting only of eye de-pigmentation. The more fragile *Drosophila* specimens were more greatly affected, and their eyes show signs of partial collapse. The more fragile fruit fly specimens also showed signs of greater mechanical damage due to handling.

Our extraction procedure produced substantial DNA yields in all samples, while minimally damaging the specimens. In ants, the only visible damage was a loss of pigmentation in the eyes. In flies, which were much more fragile, the most substantial damage came from specimen handling, such as folding of the wings. However, fly specimens also showed partial collapse of compound eyes. Representative specimens photographed before and after extraction can be seen in Figure 3.

### Sequencing, mapping and phylogenetics

**Figure 4:**
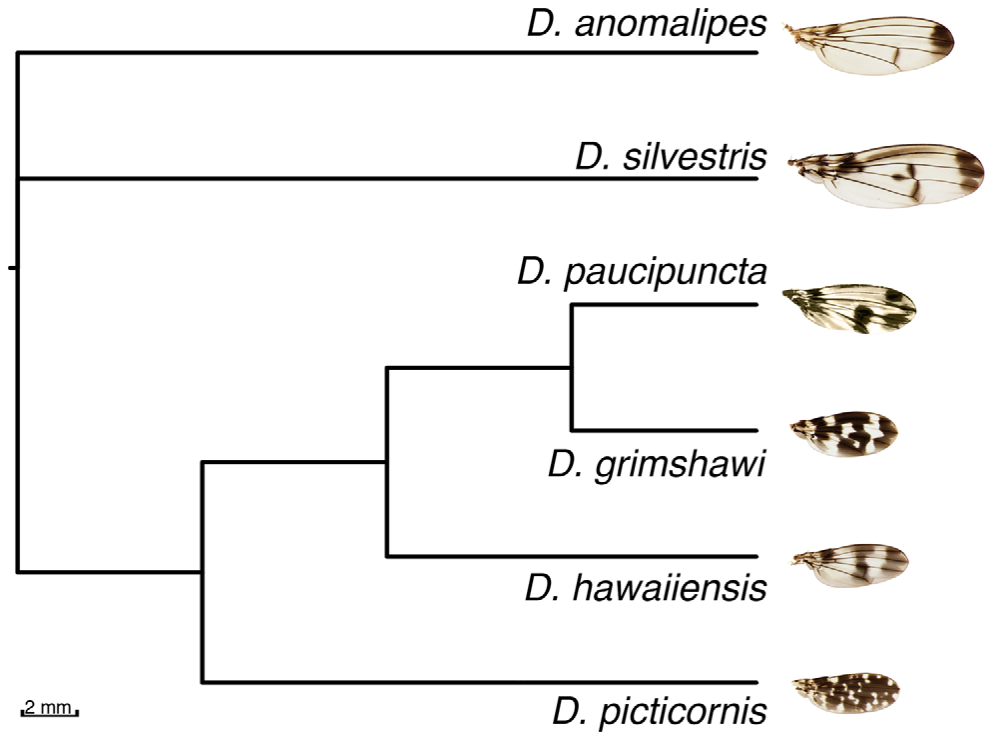
Bayesian consensus cladogram of Hawaiian fruit flies. The topology is consistent with molecular and karyotypic studies [40]–[42], and all nodes were resolved with 1.0 posterior probability. None of the previous molecular studies could resolve all of the nodes. Wing photographs from [45], except *D. paucipuncta*, which is from the present study.

Statistics on sequencing, mapping and duplication levels in each library can be found in Table 1. The mean fragment size for mapped reads was 30±2.8 bp for ant RAD tags and 48.8±5.0 bp for fly genomic shotgun reads. In the case of the fruit fly reads, this number was close to the mean fragment size of the DNA extracts (Figure 2). The mean coverage of the fruit fly genomes was 0.37±0.42 per base, ranging from 0.08 in *D. paucipuncta* to 1.0 in *D. silvestris*. The mapping rates and final coverage were not related to phylogenetic distance from the reference, and were likely more influenced by the condition of the starting DNA. The MrBayes analysis of 744 parsimony informative sites analysis quickly converged on a solution, and the average standard deviation of split frequencies was zero at the end of the analysis, which was sufficient to fully resolve the fruit fly phylogeny (Figure 4). For the ants genotyped we found 164, 16 and 1,275 SNPs in *C. floridanus, L. humile* and in *P. barbatus*, respectively.

## Discussion

We have successfully applied two powerful library preparation techniques to a diverse range of old museum-preserved specimens. Unlike commercially available kits, our library preparation procedure does not lose information due to 5’ exonuclease activity required for end repair, and works with a wide range of input DNA concentrations (Table 1). Of the 11 samples assayed, there was not a single one that failed for technical reasons, suggesting that the procedure is highly robust. Until now, small museum specimens, such as insects, were largely intractable to molecular techniques, except for amplification of short, targeted genomic regions. For instance, mapping of short reads across a species complex, and subsequent phylogenetic analysis, would have been difficult using currently available techniques, except perhaps the single-stranded protocol of Gansauge and Meyer (2013), which is probably too expensive and labor-intensive to use on more than a few samples at once. Furthermore, we were able to use a much lower number of amplification cycles (17 *vs.* 40), which should, in principle, reduce the number of PCR duplicates and errors. With more input material, it should be possible to reduce the number of cycles even further in our protocol, possibly eliminating it altogether. However this is not possible in the other protocol, which is limited to a narrow range of input DNA concentrations [15]. In general, our protocol is complementary to that of Gansauge and Meyer, which is expensive and laborious, but excels at amplifying trace amounts of material from small numbers of precious samples, whereas our protocol is rapid and robust, and thus ideally suited for processing large numbers of museum samples, from which more DNA is typically available. However, future work should compare the actual performance of the two approaches.

It is difficult to compare the performance of our extraction protocol with previously published approaches to non-destructively extracting insect DNA, because they all use different taxa [4], [5], [10]. In our study, we found that the while all specimens yielded adequate amounts of DNA, the extent to which our extraction procedure preserved specimen morphology varied between ants and flies. Ants remained largely intact, except for some discoloration of the eyes. The same procedure caused partial collapse of larger and more delicate fruit fly eyes (Figure 3). It may be possible to adjust the extraction procedure, for instance decreasing the incubation time or temperature to reduce compound eye damage. Mechanical damage associated with handling decades-old insects from pins and subsequent re-mounting, was the next major source of concern. However, while this source of damage cannot be completely avoided, but can potentially be mitigated with additional training of the staff handling the specimens. Alternatively, since the downstream library procedure is independent of the extraction protocol, other approaches may be tried, depending on the sample type [4], [5]. Future researchers may wish to compare the suitability the available protocols for their specific study system, particularly when dealing with soft-bodied specimens.

With this procedure, particularly the RAD-tag based protocol, a significant fraction of the sequence data were unusable, containing inserts that were too short for accurate genome mapping, or consisting of duplicated sequences. In the future, more precise size selection, for instance, using gel extraction, should be able to greatly decrease the amount of wasted sequence. Also, optimization of the protocol to decrease the number of PCR cycles required should yield a greater number of unique sequences. Both of these problems were reported using another recently published single-stranded protocol for sequencing degraded DNA, and suggest obvious targets for optimization [14]. However, our ongoing work suggests that phosphatase treatment prior to riboguanidine tailing of dramatically improves yield [36] (Tin and Mikheyev, unpublished). Likewise, an error-checking polymerase during second strand synthesis may also improve the performance of this protocol by reducing error rates.

Purification steps in our protocol are performed on beads, taking advantage of solid phase, reversible immobilization. This allows the protocol to be automated and carried out in bulk, which would be desirable for large-scale population studies. We routinely employ a BioMek robot (Beckman) for DNA extraction and purification steps. High-throughput sample processing allows shotgun sequencing or RAD tags to provide better quality, whole-genome data to eventually supplant currently popular, but controversial, mtDNA-based barcoding methods [37], [38]. These protocols should work even with environmentally collected samples containing degraded DNA as starting material, where amplification of a single gene several hundred bases long (such as our samples) may be impossible. RAD tags have already shown great utility in separating closely related species complexes [21], a major problem for DNA barcoding, and seem likely to replace this technology in the near future.

We were able to map raw reads for flies despite considerable phylogenetic divergence between species. In fact, mapping seemed to be more a factor of library quality than phylogenetic distance (Table 1). For instance, a lower bound on actual divergence between species in our sample can be obtained from the divergence date between *D. silvestris* and *D. picticornis*. They reside within the same species group, and diverged around six million years ago [39]. Despite low coverage (0.37±0.42 per pase) were able to fully reconstruct the fruit fly phylogeny (Figure 4). The topology corresponds that of Carson and Kaneshiro’s classic study based on chromosomal inversions [40], and more recent molecular phylogenies [41], [42]. The earlier molecular studies, however, were unable to resolve the all of the species relationships with confidence. Although the coverage appears low, a recent study has found that, for population genomics, 1 × coverage yields optimal results, balancing between tradeoffs between the amount of information gathered and sequencing effort [43].

RAD-tag markers are becoming increasingly popular for population genetic and mapping studies [19]. These markers work by focusing sequencing to particular locations in the genome anchored at restriction sites. However, as sequencing costs continue to decline and sequencing accuracy increases, reduced representation-based approaches will likely lose some of their current appeal, since the same kinds of information, and more, can be extracted using low-coverage sequence. Furthermore, when dealing with degraded data, subsequent digestion by restriction enzymes causes many fragments too short to be useful. In our study, we could obtain relatively few SNPs for most of the species. In subsequent work, we were able to increase the number of SNPs considerably, *e.g.*, though the use of a phosphatase (see above, Tin and Mikheyev, unpublished). We believe that further optimization of reaction conditions in the RAD-tag protocol may produce better results. The prospect of using low coverage shotgun sequence data to reconstruct phylogenies opens exciting opportunities for this line of research. With decreasing costs of sequencing, it should be possible to increase the number of samples, if they are available, to conduct low-coverage re-sequencing of populations for population genetic studies. However, RAD-tag markers may be workable for badly degraded DNA, where other genotyping approaches targeting longer genomic regions, such as mitochondrial or microsatellite markers, may fail.

In phylogenetics, genome size is a limiting factor for low-coverage shotgun sequencing. With increasing genome size, chances of overlap between randomly sampled fragments decrease, reducing the amount of phylogenetic information available. In the near future, with cheaper sequencing, this limitation will become less significant for population genetic and phylogenetic studies. In practice, the availability of a closely related reference genome will have a greater influence on the success of mapping, and the evolutionary distance to this genome will have to be determined empirically. In our experiment, although the sample size was not high enough to draw definitive conclusions, the mapping rate in fruit flies was not correlated with phylogenetic distance, as the reference genome species and its most distant relative had the two highest mapped read percentages. This implies that a single reference genome may be used to study the radiation of an entire clade, suggesting cost-effective strategies for future phylogenetic studies.

## References

[1] Suarez AV, Tsutsui ND (2004) The value of museum collections for research and society. BioScience 54: 66–74.

[2] Graham CH, Ferrier S, Huettman F, Moritz C, Peterson AT (2004) New developments in museum-based informatics and applications in biodiversity analysis. Trends Ecol Evol 19: 497–503 10.1016/j.tree.2004.07.006 16701313

[3] Shaffer HB, Fisher RN, Davidson C (1998) The role of natural history collections in documenting species declines. Trends Ecol Evol 13: 27–30. 21238186 10.1016/s0169-5347(97)01177-4

[4] Porco D, Rougerie R, Deharveng L, Hebert P (2010) Coupling non-destructive DNA extraction and voucher retrieval for small soft-bodied arthropods in a high-throughput context: the example of collembola. Mol Ecol Res 10: 942–945 10.1111/j.1755-0998.2010.2839.x 21565103

[5] Gilbert MTP, Moore W, Melchior L, Worobey M (2007) DNA extraction from dry museum beetles without conferring external morphological damage. PLoS ONE 2: e272 10.1371/journal.pone.0000272.t001 17342206 PMC1803022

[6] Rohland N, Siedel H, Hofreiter M (2004) Nondestructive DNA extraction method for mitochondrial DNA analyses of museum specimens. BioTechniques 36 814–6–818–21. 10.2144/04365ST05 15152601

[7] Hunter SJ, Goodall TI, Walsh KA, Owen R, Day JC (2008) Nondestructive DNA extraction from blackflies (Diptera: Simuliidae): retaining voucher specimens for DNA barcoding projects. Mol Ecol Res 8: 56–61 10.1111/j.1471-8286.2007.01879.x 21585718

[8] Favret C (2005) A new non-destructive DNA extraction and specimen clearing technique for aphids (Hemiptera). Proc Entomol Soc Wash 107: 469–470.

[9] Mikheyev A, Bresson S, Conant P (2009) Single-queen introductions characterize regional and local invasions by the facultatively clonal little fire ant *Wasmannia auropunctata* . Mol Ecol 18: 2937–2944. 19538342 10.1111/j.1365-294X.2009.04213.x

[10] Thomsen PF, Elias S, Gilbert MTP, Haile J, Munch K, et al (2009) Non-destructive sampling of ancient insect DNA. PLoS ONE 4: e5048 10.1371/journal.pone.0005048.t001 19337382 PMC2660418

[11] Andersen JC, Mills NJ (2012) DNA extraction from museum specimens of parasitic Hymenoptera. PLoS ONE 7: e45549. 23077493 10.1371/journal.pone.0045549 PMC3471897

[12] Reiss RA (2006) Ancient DNA from ice age insects: proceed with caution. Quaternary Sci Rev 25: 1877–1893.

[13] Rowe KC, Singhal S, Macmanes MD, Ayroles JF, Morelli TL, et al (2011) Museum genomics: low-cost and high-accuracy genetic data from historical specimens. Mol Ecol Res 11: 1082–1092 10.1111/j.1755-0998.2011.03052.x 21791033

[14] Meyer M, Kircher M, Gansauge M-T, Li H, Racimo F, et al (2012) A high-coverage genome sequence from an archaic Denisovan individual. Science 338: 222–226 10.1126/science.1224344 22936568 PMC3617501

[15] Gansauge M-T, Meyer M (2013) Single-stranded DNA library preparation for the sequencing of ancient or damaged DNA. Nat Protoc 8: 737–748 10.1038/nprot.2013.038 23493070

[16] Li TW, Weeks KM (2006) Structure-independent and quantitative ligation of single-stranded DNA. Anal Biochem 349: 242–246 10.1016/j.ab.2005.11.002 16325753

[17] Miller M, Dunham J, Amores A, Cresko W, Johnson E (2007) Rapid and cost-effective polymorphism identification and genotyping using restriction site associated DNA (RAD) markers. Genome Res 17: 240. 17189378 10.1101/gr.5681207 PMC1781356

[18] Baird NA, Etter PD, Atwood TS, Currey MC, Shiver AL, et al (2008) Rapid SNP discovery and genetic mapping using sequenced RAD markers. PLoS ONE 3: e3376 10.1371/journal.pone.0003376 18852878 PMC2557064

[19] Davey JW, Hohenlohe PA, Etter PD, Boone JQ, Catchen JM, et al (2011) Genome-wide genetic marker discovery and genotyping using next-generation sequencing. Nat Rev Genet 12: 499–510 10.1038/nrg3012 21681211

[20] McCormack JE, Hird SM, Zellmer AJ, Carstens BC, Brumfield RT (2013) Applications of next-generation sequencing to phylogeography and phylogenetics. Mol Phylogenet Evol 66: 526–538 10.1016/j.ympev.2011.12.007 22197804

[21] Wagner CE, Keller I, Wittwer S, Selz OM, Mwaiko S, et al (2013) Genome-wide RAD sequence data provide unprecedented resolution of species boundaries and relationships in the Lake Victoria cichlid adaptive radiation. Mol Ecol 22: 787–798 10.1111/mec.12023 23057853

[22] Folmer O, Black M, Hoeh W, Lutz R, Vrijenhoek R (1994) DNA primers for amplification of mitochondrial cytochrome c oxidase subunit I from diverse metazoan invertebrates. Mol Marine Biol Biotechnol 3: 294–299. 7881515

[23] Jansen G, Savolainen R, Vepsäläinen K (2009) DNA barcoding as a heuristic tool for classifying undescribed Nearctic Myrmica ants (Hymenoptera: Formicidae). Zoologica Scripta 38: 527–536.

[24] Yue D, Tabor S, Nichols NM (2008) Template-independent DNA polymerases. Curr Protoc Mol Biol. Wiley Online Library. doi:10.1002/0471142727.mb0306s84. 10.1002/0471142727.mb0306s84 18972388

[25] Martin M (2011) Cutadapt removes adapter sequences from high-throughput sequencing reads. EMBnet journal 17: 10–12.

[26] Langmead B, Salzberg SL (2012) Fast gapped-read alignment with Bowtie 2. Nature Methods 9: 357–359 10.1038/nmeth.1923 22388286 PMC3322381

[27] Smith CR, Smith CD, Robertson HM, Helmkampf M, Zimin A, et al (2011) Draft genome of the red harvester ant *Pogonomyrmex barbatus* . PNAS 108: 5667–5672 10.1073/pnas.1007901108 21282651 PMC3078412

[28] Smith CD, Zimin A, Holt C, Abouheif E, Benton R, et al. (2011) Draft genome of the globally widespread and invasive Argentine ant (*Linepithema humile*). PNAS. doi:10.1073/pnas.1008617108. 10.1073/pnas.1008617108 PMC3078359 21282631

[29] Bonasio R, Zhang G, Ye C, Mutti NS, Fang X, et al (2010) Genomic comparison of the ants *Camponotus floridanus* and *Harpegnathos saltator* . Science 329: 1068–1071 10.1126/science.1192428 20798317 PMC3772619

[30] Stark A, Lin MF, Kheradpour P, Pedersen JS, Parts L, et al (2007) Discovery of functional elements in 12 Drosophila genomes using evolutionary signatures. Nature 450: 219–232. 17994088 10.1038/nature06340 PMC2474711

[31] Auwera GA, Carneiro MO, Hartl C, Poplin R, del Angel G, et al.. (2013) From FastQ data to high-confidence variant calls: the Genome Analysis Toolkit Best practices pipeline. Curr Protoc Bioinformatics: 11.10.1–11.10.33. 10.1002/0471250953.bi1110s43 PMC4243306 25431634

[32] Li H, Handsaker B, Wysoker A, Fennell T, Ruan J, et al (2009) The sequence alignment/map format and SAMtools. Bioinformatics 25: 2078–2079 10.1093/bioinformatics/btp352 19505943 PMC2723002

[33] Smit A, Hubley R, Green P. *RepeatMasker Open-3.0* Available: http://www.repeatmasker.org.

[34] Quinlan AR, Hall IM (2010) BEDTools: a flexible suite of utilities for comparing genomic features. Bioinformatics 26: 841–842 10.1093/bioinformatics/btq033 20110278 PMC2832824

[35] Ronquist F, Huelsenbeck JP (2003) MrBayes 3: Bayesian phylogenetic inference under mixed models. Bioinformatics 19: 1572–1574. 12912839 10.1093/bioinformatics/btg180

[36] Zimmermann J, Hajibabaei M, Blackburn DC, Hanken J, Cantin E, et al (2008) DNA damage in preserved specimens and tissue samples: a molecular assessment. Frontiers in Zoology 5: 18 10.1186/1742-9994-5-18 18947416 PMC2579423

[37] Waugh J (2007) DNA barcoding in animal species: progress, potential and pitfalls. Bioessays 29: 188–197 10.1002/bies.20529 17226815

[38] Hebert PDN, Penton EH, Burns JM, Janzen DH, Hallwachs W (2004) Ten species in one: DNA barcoding reveals cryptic species in the neotropical skipper butterfly *Astraptes fulgerator* . PNAS 101: 14812–14817 10.1073/pnas.0406166101 15465915 PMC522015

[39] Bonacum J, O'Grady PM, Kambysellis M, Desalle R (2005) Phylogeny and age of diversification of the planitibia species group of the Hawaiian Drosophila. Mol Phylogenet Evol 37: 73–82 10.1016/j.ympev.2005.03.008 16182150

[40] Carson HL, Kaneshiro KY (1976) Drosophila of Hawaii: systematics and ecological genetics. Annu Rev Ecol Syst 7: 311–345.

[41] O'Grady PM, Baker RH, Durando CM, Etges WJ, DeSalle R (2001) Polytene chromosomes as indicators of phylogeny in several species groups of Drosophila. BMC Evol Biol 1: 6. 11696235 10.1186/1471-2148-1-6 PMC59584

[42] O'Grady PM, Lapoint RT, Bonacum J, Lasola J, Owen E, et al (2011) Phylogenetic and ecological relationships of the Hawaiian Drosophila inferred by mitochondrial DNA analysis. Mol Phylogenet Evol 58: 244–256 10.1016/j.ympev.2010.11.022 21144904

[43] Alex Buerkle C, Gompert Z (2013) Population genomics based on low coverage sequencing: how low should we go? Mol Ecol 22: 3028–3035 10.1111/mec.12105 23174005

[44] Schmidt WM, Mueller MW (1996) Controlled ribonucleotide tailing of cDNA ends (CRTC) by terminal deoxynucleotidyl transferase: a new approach in PCR-mediated analysis of mRNA sequences. Nucleic Acids Res 24: 1789–1791. 8650002 10.1093/nar/24.9.1789 PMC145852

[45] Edwards KA, Doescher LT, Kaneshiro KY, Yamamoto D (2007) A Database of Wing Diversity in the Hawaiian Drosophila. PLoS ONE 2: e487 10.1371/journal.pone.0000487.t001 17534437 PMC1872047

